# Histone deacetylase 4 promotes type I interferon signaling, restricts DNA viruses, and is degraded via vaccinia virus protein C6

**DOI:** 10.1073/pnas.1816399116

**Published:** 2019-05-24

**Authors:** Yongxu Lu, Jennifer H. Stuart, Callum Talbot-Cooper, Shuchi Agrawal-Singh, Brian Huntly, Andrei I. Smid, Joseph S. Snowden, Liane Dupont, Geoffrey L. Smith

**Affiliations:** ^a^Department of Pathology, University of Cambridge, CB2 1QP Cambridge, United Kingdom;; ^b^Cambridge Institute for Medical Research, University of Cambridge, CB2 0XY Cambridge, United Kingdom

**Keywords:** type I interferon signaling, STAT2 recruitment, histone deactylase 4, vaccinia virus protein C6, immune evasion

## Abstract

Interferons (IFNs) represent an important host defense against viruses. Type I IFNs induce JAK-STAT signaling and expression of IFN-stimulated genes (ISGs), which mediate antiviral activity. Histone deacetylases (HDACs) perform multiple functions in regulating gene expression and some class I HDACs and the class IV HDAC, HDAC11, influence type I IFN signaling. Here, HDAC4, a class II HDAC, is shown to promote type I IFN signaling and coprecipitate with STAT2. Pharmacological inhibition of class II HDAC activity, or knockout of HDAC4 from HEK-293T and HeLa cells, caused a defective response to IFN-α. This defect in HDAC4^−/−^ cells was rescued by reintroduction of HDAC4 or catalytically inactive HDAC4, but not HDAC1 or HDAC5. ChIP analysis showed HDAC4 was recruited to ISG promoters following IFN stimulation and was needed for binding of STAT2 to these promoters. The biological importance of HDAC4 as a virus restriction factor was illustrated by the observations that (*i*) the replication and spread of vaccinia virus (VACV) and herpes simplex virus type 1 (HSV-1) were enhanced in HDAC4^−/−^ cells and inhibited by overexpression of HDAC4; and (*ii*) HDAC4 is targeted for proteasomal degradation during VACV infection by VACV protein C6, a multifunctional IFN antagonist that coprecipitates with HDAC4 and is necessary and sufficient for HDAC4 degradation.

## Significance

Histone deacetylases (HDACs) are regulators of host gene expression. HDAC4 is shown here to have an important role in type I interferon (IFN) signaling. Here, multiple cell lines lacking HDAC4 had impaired responses to IFN-α and were rescued by reintroduction of HDAC4. The biological significance of HDAC4 was demonstrated by the enhanced replication and spread of two DNA viruses, vaccinia virus (VACV) and herpes simplex virus type I, in HDAC4^−/−^ cells, and their diminished replication when HDAC4 was overexpressed. Furthermore, HDAC4 was targeted for proteasomal degradation early after infection with VACV, and VACV protein C6, an inhibitor of type I IFN signaling, was necessary and sufficient for this degradation. In summary, HDAC4 is a restriction factor for large DNA viruses.

Histone deacetylases (HDACs) can repress gene expression by deacetylating histones leading to strengthened histone–DNA interactions and chromatin condensation (1). HDACs can also induce gene expression; for instance, in leukemic cells, similar numbers of genes are up- or down-regulated by the broad spectrum HDAC inhibitor trichostatin A (TSA) (2). Furthermore, >1,750 proteins may be acetylated, suggesting acetylation has important regulatory functions beyond histone modification (3). Aberrant HDAC expression or function is linked to several human diseases and cancers, and HDAC inhibitors have potential as anticancer therapeutics (4).

In humans there are 18 HDACs that are classified in four subfamilies: class I (HDACs 1, 2, 3, and 8); class II HDACs, subdivided into class IIa (HDACs 4, 5, 7, and 9) and class IIb (HDACs 6 and 10); class III (sirtuins 1–7); and class IV (HDAC11) (1, 4).

Type I interferons (such as IFNα/β) are secreted glycoproteins that induce antimicrobial states in cells, orchestrate the inflammatory response to invading pathogens, and activate the adaptive immune system (5, 6). HDAC activity is important for signaling leading to IFN expression because this is blocked by TSA (7). HDAC6 (8, 9), HDAC2 (10), and HDAC9 (11) were each reported to promote type I IFN expression. For HDAC3, different studies using different cell types reported either activation (12) or repression (13) of IFN-β expression. On the other hand, HDAC4 (14), HDAC1, and HDAC8 (8, 15) were reported to repress type I IFN expression.

HDAC activity also affects type I IFN-stimulated gene (ISG) expression as shown by impairment of ISG transcription by addition of TSA (16) or the absence of HDAC1 (17, 18), HDAC2 (17), or HDAC3 (19). HDAC1 coprecipitates with signal transducer and activator of transcription 1 (STAT1) and STAT2 (18). Conversely, HDAC11 negatively regulates type I IFN signaling by controlling lysine fatty acylation of serine hydroxymethyltransferase 2 (SHMT2) and thereby the surface level of type I IFN receptor 1 (20). HDAC1, HDAC2, and HDAC3 are also required for type II IFN signaling (21).

Consistent with a role in innate immunity, HDACs can influence the outcome of virus infection and consequently, some viruses have evolved proteins to counteract or hijack HDAC activity to their advantage. For example, pharmacological inhibition of HDACs induced reactivation of quiescent genomes of Kaposi sarcoma-associated herpesvirus (KSHV) and Epstein-Barr virus (EBV) (22–24). Similarly, HDAC inhibitors vorinostat and valproic acid disrupt HIV-1 latency in CD4^+^ T cells (25, 26). During herpes simplex virus type 1 (HSV-1) infection, HDAC1 binds to the corepressor for element-1–silencing transcription factor/RE1 silencing transcription factor (CoREST/REST) complex to inhibit viral gene expression, and this is countered by HSV-1 protein ICP0 that dissociates HDAC1 from the viral genome (27). In human cytomegalovirus (HCMV) infection the immediate early (IE) viral proteins IE1 and IE2 function to antagonize HDAC3, thereby promoting viral replication (28, 29). Interestingly, IE1 also associates with HDAC1 and hijacks its function to repress IE gene expression, thereby promoting early and late viral gene expression (29).

Although interplay between viruses and class I HDACs is well established, less is known about viral interactions with members of the class II HDAC family, such as HDAC4. HSV-1 protein ICP0 was reported to coprecipitate with HDAC4, however, the functional consequence of this is unknown (30). EBV nuclear antigen leader protein (EBNA-LP) also coprecipitates with HDAC4 and overexpression of HDAC4 reduced reporter gene expression driven by an EBNA-LP–responsive promoter (31). Additionally, in EBV-infected B lymphocytes, HDAC4 is recruited by its interaction partner myocyte enhancer factor 2 (MEF2) to the EBV genome, and HDAC4 overexpression restricts gene transcription at the EBV immediate early gene BZLF1 promoter (32). Recently, it was reported that knockdown of HDAC4 restricted IRF3 phosphorylation (14) and HDAC4 knockout reduced HSV-1 replication (33).

This study reports that HDAC4 is required for type I IFN signaling and restricts the replication of HSV-1 and vaccinia virus (VACV). Mechanistically, HDAC4 coprecipitates with STAT2 and is recruited to IFN-stimulated response element (ISRE)-containing promoters following addition of type I IFN. Without HDAC4, binding of STAT2 to these promoters after IFN-α addition is greatly reduced. Lastly, HDAC4 is targeted for proteasomal degradation during VACV infection. VACV expresses many inhibitors of innate immunity and IFN signaling (34, 35) and one of these, called C6, is a multifunctional IFN antagonist (36, 37). Here, C6 is shown to coprecipitate with HDAC4 and to be necessary and sufficient for inducing the proteasomal degradation of HDAC4. The targeting of HDAC4 by VACV provides biological evidence of the importance of HDAC4 as a viral restriction factor.

## Results

### HDAC Inhibitors Block Type I IFN Signaling.

**Fig. 1.**
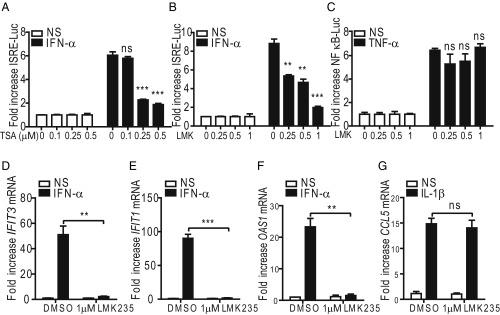
HDAC inhibitors TSA and LMK235 (LMK) inhibit the response to IFN-α. (*A*) HeLa cells seeded in 96-well plates were cotransfected in triplicate with 100 ng per well ISRE-luciferase reporter plasmid and 10 ng per well Renilla luciferase plasmid overnight. Cells were then treated simultaneously with 250 nM TSA and 1,000 units/mL IFN-α for 6 h. After cytokine stimulation, a cell lysate was harvested and firefly luciferase was measured and normalized to Renilla luciferase control. The fold induction of firefly luciferase is shown relative to unstimulated controls. (*B* and *C*) LMK235 inhibits the response to IFN-α but not IL-1β. HeLa cells were transfected as in *A* except in *C* where an NF-κB-luciferase reporter was used. Cells were then treated simultaneously with the indicated concentrations of LMK235 and 1,000 units/mL IFN-α or 100 μg/μL IL-1β for 6 h. The fold increase in firefly luciferase expression, normalized to Renilla control, is presented as in *A*. (*D*–*G*) LMK235 inhibits ISG expression in response to IFN-α. HeLa cells were treated simultaneously with 1 μM LMK235 or DMSO and 1,000 units/mL IFN-α (*D*–*F*) or 100 ng/mL IL-1β (*G*) for 6 h. mRNA was extracted from cells and used for RT-qPCR analysis. Data are presented as the fold induction of mRNA expression relative to the unstimulated, DMSO-treated control and relative to *GAPDH* mRNA expression. Data shown are representative of three independent experiments. ns = not significant, *P ≤ 0.05, **P ≤ 0.01, ***P ≤ 0.001.

To investigate further the roles of HDACs in type I IFN signaling, the effect of TSA on type I ISRE-dependent reporter gene expression was assessed in HeLa cells. Consistent with previous reports, type I IFN signaling was largely inhibited by TSA (Fig. 1*A*). Next, an inhibitor of class II HDACs, LMK235, was used and found to also inhibit IFN-α–induced ISRE-dependent gene expression in a dose-dependent manner (Fig. 1*B*). In contrast, LMK235 treatment had no effect on NF-κB–dependent luciferase expression in response to TNF-α stimulation (Fig. 1*C*). These data indicate that class II HDACs have a function in type I IFN signaling.

To explore this further, the effect of LMK235 on endogenous gene expression in response to IFN-α and IL-1β was analyzed in HeLa cells by reverse transcription-quantitative PCR (RT-qPCR). LMK235 inhibited the induction of mRNA of three IFN-α–responsive genes [*IFIT3, IFIT1*, and *2′5′-OAS* (*OAS1*)] (Fig. 1 *D*–*F*) but not *CCL5* and *IL-6* that are NF-κB–dependent genes induced by IL-1β (Fig. 1*G* and *SI Appendix*, Fig. S1). These data indicate that class II HDAC activity is needed for ISRE-dependent gene expression in response to IFN-α but not for the expression of IL-1β–induced genes that require NF-κB activation.

### HDAC4 Is Important for Type I IFN-Stimulated Gene Expression.

A possible role for HDAC4 in type I IFN signaling was investigated using HDAC4^−/−^ cell lines generated by CRISPR/Cas9 genome editing (38). Two clonal HDAC4^−/−^ HEK-293T cell lines, derived using different guide RNAs (gRNAs), and two HDAC4^−/−^ HeLa cell lines, genome edited with gRNA1, were selected as described in *SI Appendix*, Fig. S2*A*. Sequencing of the genomic region targeted by the gRNAs confirmed frameshift mutations had been introduced into each allele and that no wild-type (WT) allele remained (*SI Appendix*, Fig. S2 *B* and *C*). Consistent with this, immunoblotting showed loss of HDAC4 expression (*SI Appendix*, Fig. S3). These cell lines showed no significant difference in growth rate compared with the parental cell lines, indicating that although HDAC4^−/−^ mice displayed skeletal defects and a very limited life span (39), HDAC4 is nonessential for human cell lines in vitro.

**Fig. 2.**
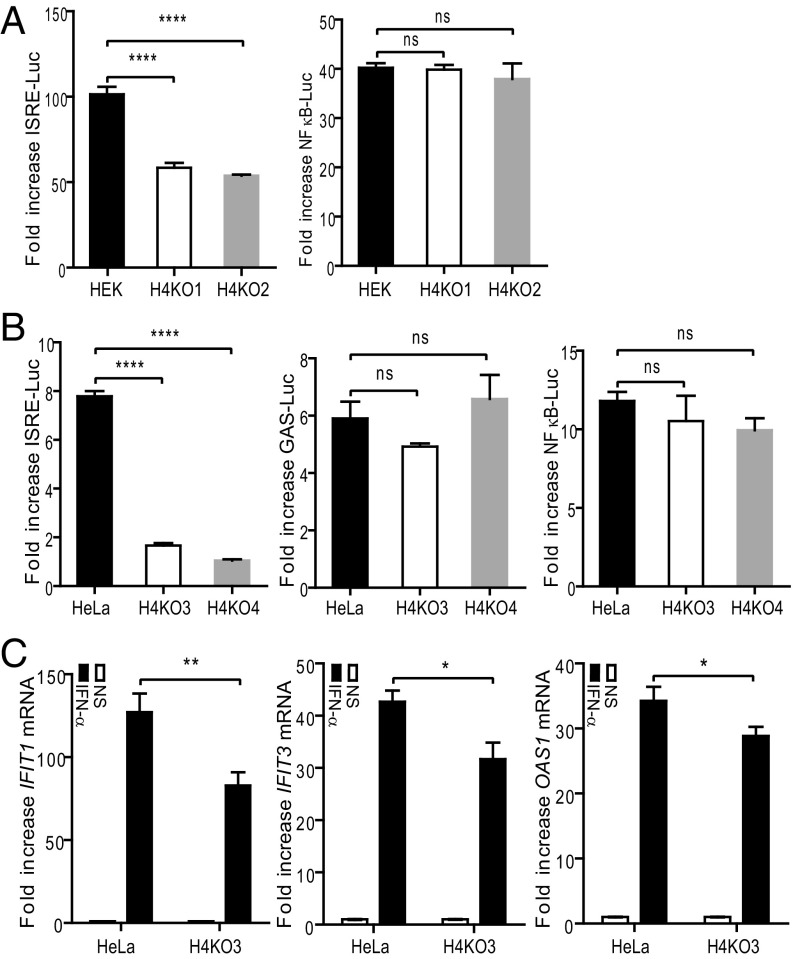
HDAC4^−/−^ cells respond poorly to IFN-α. (*A*) HDAC4^−/−^ HEK-293T clones H4KO1 and H4KO2 and parental HEK-293T cells were cotransfected in triplicate with 100 ng per well ISRE-luciferase plasmid (*Left*) or NF-κB–luciferase plasmid (*Right*) and 10 ng per well Renilla luciferase plasmid overnight. Cells were then stimulated with 1,000 units/mL IFN-α or 10 ng/mL TNF-α for 6 h. Firefly luciferase activity was measured and normalized to Renilla luciferase expression and the fold induction relative to unstimulated controls is shown. (*B*) HDAC4^+/+^ HeLa, HDAC4^−/−^ clones H4KO3 and H4KO4 cells were cotransfected with 100 ng per well ISRE-luciferase (*Left*) or GAS-luciferase (*Center*) or NF-κB–luciferase (*Right*) plasmid and 10 ng per well Renilla luciferase. Transfected cells were stimulated with 1,000 units/mL IFN-α (*Left*) or 250 ng/mL IFN-γ (*Center*) or 10 ng/mL TNF-α (*Right*) for 6 h. Cell lysates were prepared and luciferase activity was measured and presented as in *A*. (*C*) RT-qPCR analysis of mRNAs. HeLa or H4KO3 cells were seeded in six-well plates at 2 × 10^6^ cells per well in triplicate. The next day, cells were stimulated with 1,000 units/mL IFN-α for 4 h or left untreated. Cellular mRNA was extracted and reverse transcribed into cDNA, then qPCR analysis was performed to quantify ISG induction. Data shown are representative of three independent experiments. ns = not significant, *P ≤ 0.05, **P ≤ 0.01, ****P ≤ 0.0001.

These HEK-293T HDAC4^−/−^ cell lines (H4KO1 and H4KO2) were then used in reporter gene assays. ISRE-luciferase expression after IFN-α stimulation was significantly diminished in H4KO1 and H4KO2 cells compared with parental HEK-293T cells (Fig. 2 *A*, *Left*), whereas the NF-κB response to TNF-α was normal (Fig. 2 *A*, *Right*). So, consistent with the results using pharmacological inhibitors of HDACs, two independent HDAC4^−/−^ cell lines showed impaired type I IFN signaling. Similar analysis in HDAC4^−/−^ HeLa cells (H4KO3 and H4KO4) showed a greater deficiency in response to IFN-α stimulation (Fig. 2 *B*, *Left*). In contrast, IFN-γ–activated sequence (GAS)-luciferase and TNF-α–induced NF-κB–luciferase were not significantly different in these HDAC4^−/−^ cell lines (Fig. 2 *B*, *Center* and *Right*). The role of HDAC4 in type I IFN signaling was also investigated by RT-qPCR of endogenous ISGs (*IFIT3, IFIT1,* and *OAS1*) in HeLa HDAC4^−/−^ cells. For each ISG there was a significant reduction in the response to IFN-α compared with control cells (Fig. 2*C*). The greater deficiency in the response to IFN measured by reporter gene assay than by RT-qPCR of endogenous genes may reflect the different methodologies used. The greater inhibition of ISG transcription by LMK235 (Fig. 1) rather than knockout of HDAC4 may reflect the inhibition of other class II HDACs by LMK235.

### HDAC4, but Not HDAC1 or HDAC5, Rescues the Type I IFN Response in HDAC4^−/−^ Cells.

**Fig. 3.**
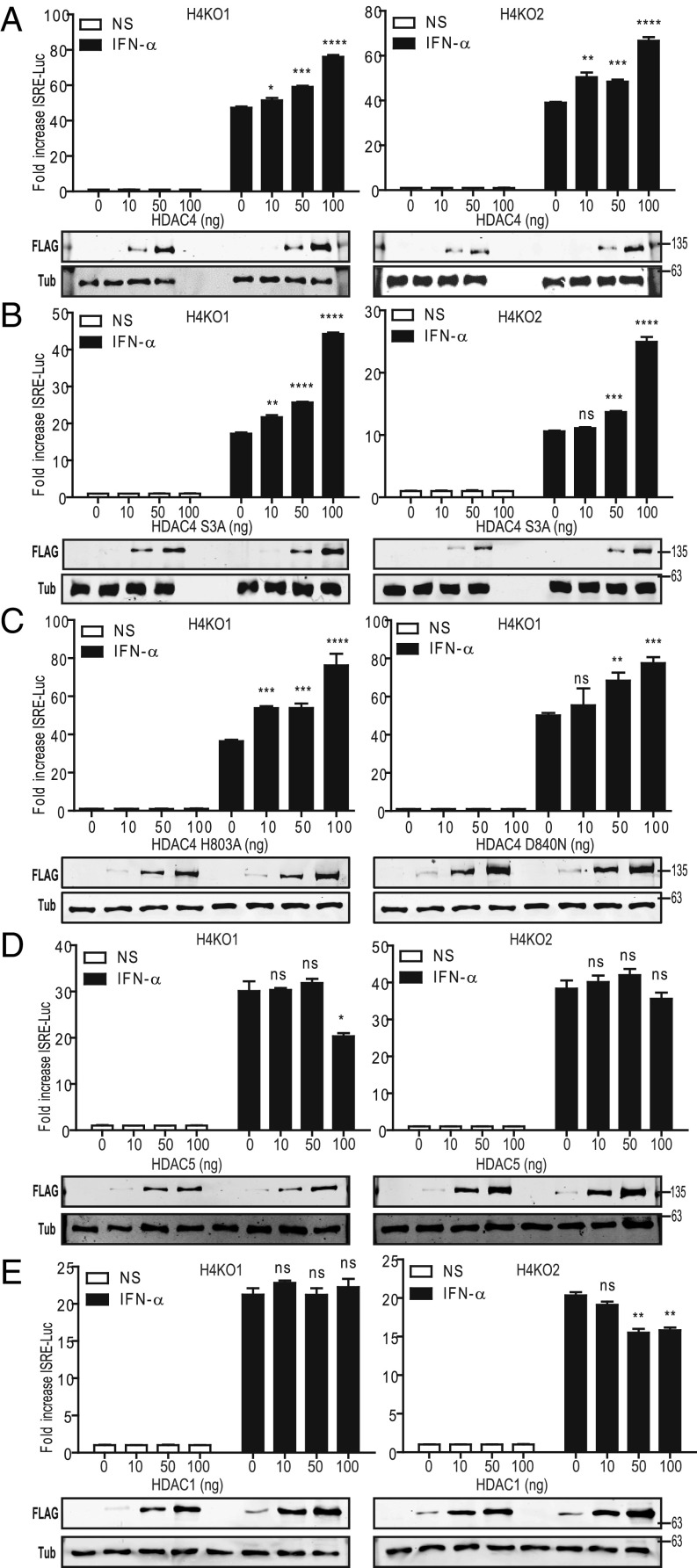
HDAC4, but not HDAC1 and HDAC5, rescues the defective IFN-α response in HDAC4^−/−^ cells. (*A*) ISRE luciferase, Renilla, and HDAC4-FLAG expression plasmids were cotransfected into H4KO1 or H4KO2 cells (as indicated) overnight. The HDAC4-FLAG expression plasmid was transfected at 0, 10, 50, or 100 ng per well. Following overnight transfection, cells were stimulated with 1,000 units/mL IFN-α for 6 h. Cell lysates were prepared and firefly luciferase expression was measured and normalized to Renilla luciferase. Results show the fold induction of firefly luciferase relative to the unstimulated controls. (*B*) Reporter gene assay as in *A* performed with HDAC4 3SA-FLAG in H4KO1 or H4KO2 cells as indicated. (*C*) Reporter gene assay as in *A* performed with HDAC4 H803A-FLAG or HDAC4 D840N-FLAG in H4KO1 cells. (*D* and *E*) Reporter gene assay as in *A* but using HDAC5 (*D*) or HDAC1 (*E*). Data shown are representative of three independent experiments. *Bottom* shows immunoblots for FLAG-tagged proteins and α-tubulin (Tub). ns = not significant, *P ≤ 0.05, **P ≤ 0.01, ***P ≤ 0.001, ****P ≤ 0.0001.

Four HDAC4^−/−^ cell lines all showed a reduced response to type I IFN. To confirm this deficiency was due to loss of HDAC4 rather than an off-target effect induced by CRISPR/Cas9, FLAG-tagged HDAC4 was expressed in two HDAC4^−/−^ cell lines (Fig. 3*A*) and WT cells (*SI Appendix*, Fig. S4), and the effect analyzed. Immunoblotting demonstrated dose-dependent FLAG-HDAC4 expression. In HDAC4^−/−^ cells there was a dose-dependent increase in ISRE-luciferase activity following type I IFN stimulation (Fig. 3*A*). However, no increase was observed in WT HEK-293T cells as HDAC4 expression increased, where higher expression was slightly inhibitory (*SI Appendix*, Fig. S4).

Next HDAC4 mutants were tested for their ability to complement for loss of HDAC4. Protein 14-3-3 interacts with HDAC4 and regulates its intracellular localization (40, 41). The interaction of 14-3-3 with HDAC4 is abolished by serine-to-alanine mutations at HDAC4 S246, S467, and S632 (HDAC4 3SA) and results in nuclear localization of HDAC4 (41). FLAG-HDAC4 3SA was introduced into HDAC4^−/−^ cells and found to complement HDAC4 deficiency as efficiently as WT HDAC4, indicating that interaction with 14-3-3 is not necessary for type I IFN signaling (Fig. 3*B*). The nuclear location of HDAC4 3SA suggests that HDAC4 promotes type I IFN signaling within the nucleus (42). To examine if HDAC4 enzymatic activity is needed for the type I IFN signaling, two HDAC4 mutants, H803A and D840N, which lack enzymatic activity and do not interact with HDAC3 (43, 44), were tested. Introduction of HDAC4 H803A and D840N into HDAC4^−/−^ cells complemented the type I IFN response as efficiently as wild type, indicating that HDAC4 enzymatic activity and the interaction with HDAC3 are not required for IFN-α signaling (Fig. 3*C*).

Expression of FLAG-HDAC5 or FLAG-HDAC1 in HDAC4^−/−^ cells did not restore IFN-α–induced gene expression and so was unable to complement loss of HDAC4 (Fig. 3 *D* and *E*).

Given that HDAC4 deacetylase activity was not needed for type I IFN signaling, the inhibition of this pathway by the HDAC inhibitor LMK235 suggests that enzymatic activity of another class II HDAC is required for type I IFN signaling. Consistent with this, LMK235 still inhibited type I FN signaling in two HDAC4^−/−^ cell lines (*SI Appendix*, Fig. S5).

### HDAC4 Is Recruited to IFN-α–Stimulated Promoters and Is Needed for STAT2 Recruitment.

**Fig. 4.**
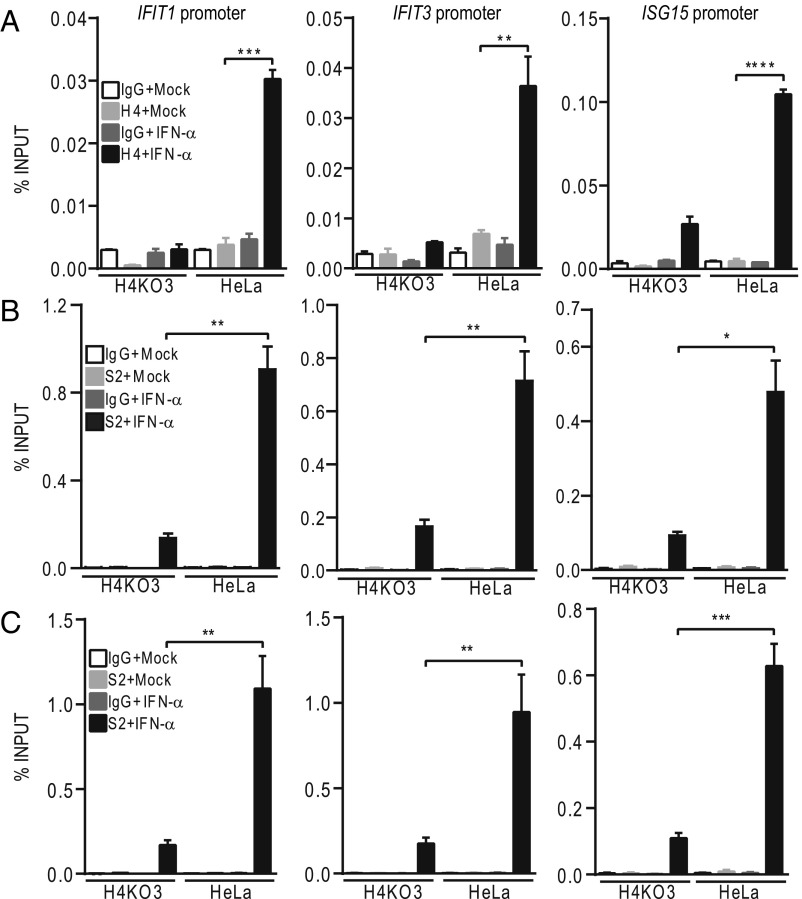
ChIP analysis of HDAC4 and STAT2 occupancy of the promoter of genes encoding *IFIT1, IFIT3*, and *ISG15*. HeLa cells or HDAC4^−/−^ cells (H4KO3) were mock treated or treated with 1,000 units/mL IFN-α for 3 h. Samples were then processed for ChIP analysis (*Materials and Methods*) using antibodies against HDAC4, H4 (Santa Cruz sc-46672) (*A*) or STAT2, S2 (*B* and *C*). The antibody used in *B* was from Cell Signaling, 72604, and the antibody used in *C* was from ACTIVE MOTIF, 61651. In each case ChIP was performed in parallel with a control IgG. Error bars denote mean ± SD of three technical replicates. Statistical analyses compare HeLa cells with or without IFN-α treatment (*A*), or HeLa cells with H4KO3 cells both with IFN-α treatment (*B* and *C*). *P ≤ 0.05, **P ≤ 0.01, ***P ≤ 0.001, ****P ≤ 0.0001.

To investigate how HDAC4 contributes to the type I IFN response, chromatin immunoprecipitation (ChIP) assays were undertaken with antibodies against HDAC4 and STAT2. HeLa and H4KO3 cell lines were treated with IFN-α and then fixed with formaldehyde to crosslink chromatin-associated proteins. After chromatin fragmentation, samples were immunoprecipitated with two antibodies against HDAC4 or STAT2 and the enriched chromatin was analyzed by qPCR. One anti-HDAC4 antibody did not work in this assay. However, as shown in Fig. 4*A*, three ISG promoters (*IFIT1, IFIT3,* and *ISG15*) were enriched by the other anti-HDAC4 antibody after IFN-α stimulation compared with mock-treated cells, and, as expected, there was no enrichment in H4KO3 cells. Similar analysis with two different anti-STAT2 antibodies showed enhanced binding of STAT2 following addition of IFN-α to WT cells, but binding of STAT2 to the IFIT1, IFIT3, and ISG15 promoters was greatly reduced in H4KO3 cells compared with WT cells (Fig. 4 *B* and *C*). Collectively, these data show that HDAC4 is recruited to ISG promoters after IFN-α stimulation and that HDAC4 is required for normal STAT2 recruitment to these promoters.

### HDAC4 Coprecipitates with STAT2 via the STAT2 Transactivation Domain.

**Fig. 5.**
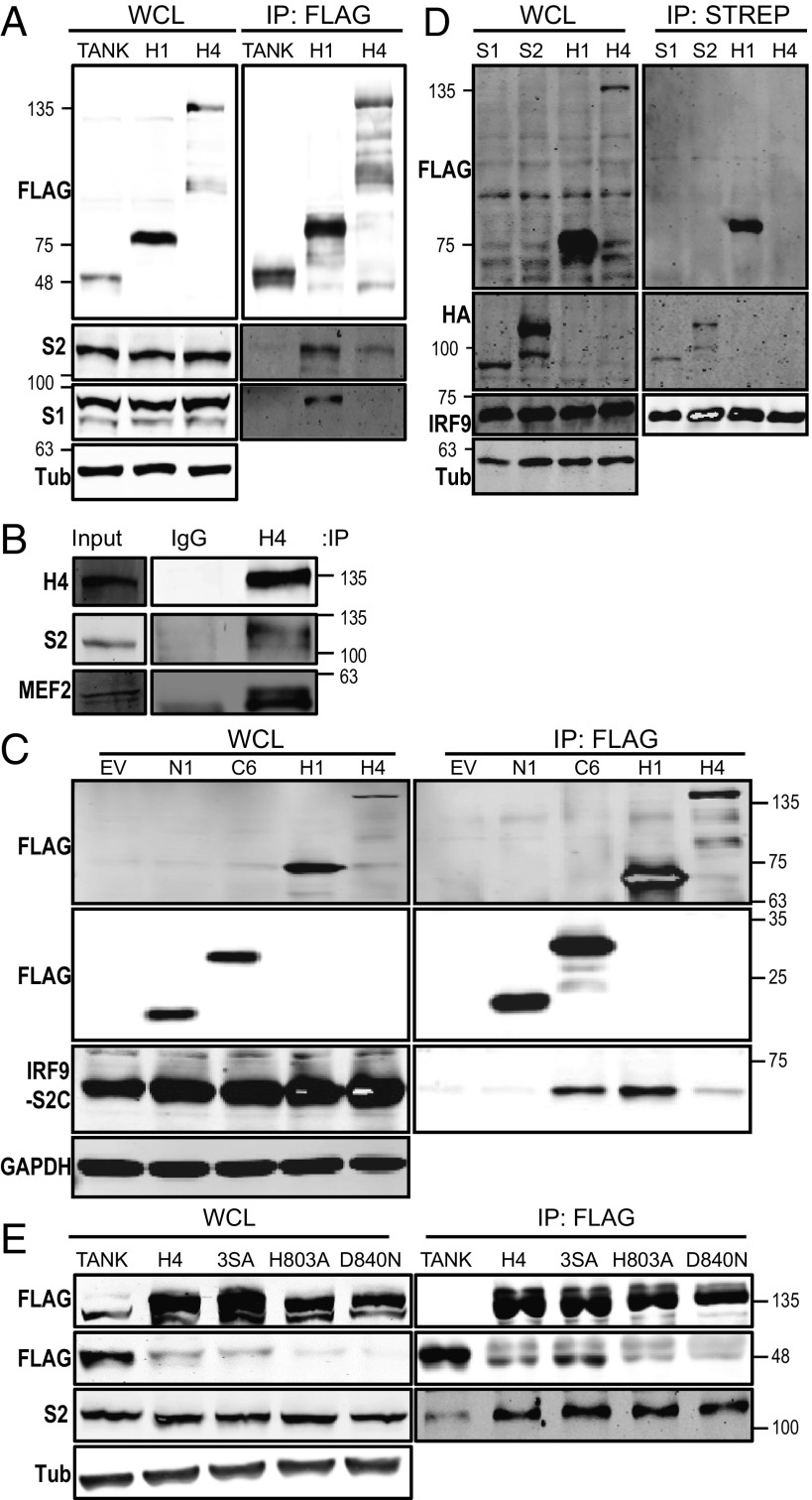
HDAC4 coprecipitates with STAT2 via its transactivation domain. (*A*) HEK-293T cells were transfected with plasmids encoding FLAG-tagged TANK, HDAC1, or HDAC4. The following day, cell lysates were prepared and proteins were immunoprecipitated via the FLAG-epitope. Inputs (*Left*) or immunoprecipitates (*Right*) were analyzed by immunoblotting with the indicated antibodies. H1, HDAC1; H4, HDAC4; S2, STAT2; S1, STAT1. (*B*) Coimmunoprecipitation of HDAC4 and STAT2 at endogenous levels from HEK-293T cells. Cell lysates collected from 1 × 10^7^ HEK-293T cells were incubated with antibody against HDAC4 or mouse IgG for 2 h. Samples were then immunoprecipitated with Protein G Sepharose overnight. H4, mouse anti-HDAC4 IgG; IgG, mouse IgG control. (*C*) HEK-293T cells were transfected with empty vector (EV), TAP-tagged C6 or N1, or FLAG-tagged HDAC1 or HDAC4. All samples were also transfected with IRF9-S2C [a plasmid encoding IRF9 fused to the last 104 aa of STAT2 (the transactivation domain)]. After 24 h transfection, cell lysates were prepared and immunoprecipitated via the FLAG-epitope. Inputs and immunoprecipitates were analyzed as described in *A*. IRF9-S2C was analyzed with antibody against STAT2 TAD. (*D*) HEK-293T cells were transfected with HA-tagged STAT1 or STAT2, FLAG-tagged HDAC1, or HDAC4. The samples were cotransfected with TAP-tagged IRF9. After 24 h transfection, cell lysates were precipitated via the streptavidin-epitope and analyzed as in *C*. (*E*). HEK-293T cells were transfected with plasmids expressing FLAG-tagged TANK, HDAC4, HDAC4 3SA, HDAC4 H803A, or HDAC4 D840N. After 24 h, cell lysates were immunoprecipitated via the FLAG-epitope and analyzed as in *C*. The positions of molecular mass markers are shown in kilodaltons on the *Left* (*A* and *D*) or *Right* (*B*, *C*, and *E*). The experiment was conducted three times and representative images are shown.

The reduced STAT2 binding to the IFN-α–stimulated promoters suggested that HDAC4 might interact with components of the ISGF3 complex (IRF9, STAT1, and STAT2) and this was investigated by immunoprecipitation. FLAG-tagged HDAC4 coprecipitated with STAT2 but not STAT1, while FLAG-tagged TANK did not coprecipitate with either STAT1 or STAT2 (Fig. 5*A*). The known interaction of HDAC1 with STAT2 served as a positive control (18). To confirm the HDAC4–STAT2 interaction occurred at endogenous levels, endogenous HDAC4 was immunoprecipitated and the immunoprecipitates were blotted for STAT2 and also MEF2, a known HDAC4-binding protein (44). This showed coprecipitation of STAT2 and MEF2, whereas this was not seen with a control IgG (Fig. 5*B*). A fusion protein containing the C-terminal 104 amino acids of STAT2, including the transactivation domain (TAD) fused to IRF9 also coprecipitated with HDAC4, indicating this association required only this region of STAT2 (Fig. 5*C*). VACV protein C6 also coprecipitated with this domain as reported (36). In contrast, TAP-tagged IRF9 did not coprecipitate with HDAC4, but coprecipitated with HA-tagged STAT1 and STAT2 and FLAG-tagged HDAC1 (Fig. 5*D*). The ability of the HDAC4 mutants used above to coprecipitate with STAT2 was also investigated. FLAG-tagged HDAC4 3SA, H803A, and D840N each retained the ability to coprecipitate endogenous STAT2 (Fig. 5*E*).

### HDAC4 Restricts VACV and HSV-1 Replication and Spread.

**Fig. 6.**
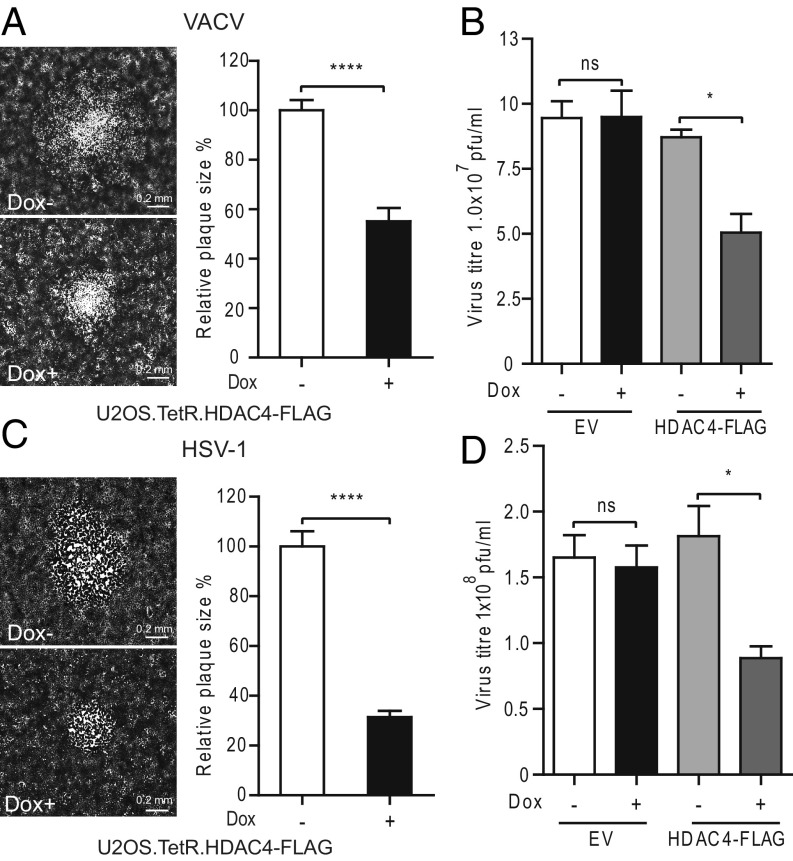
Overexpression of HDAC4-FLAG in U2OS cells restricts VACV (*A* and *B*) and HSV-1 (*C* and *D*) replication and spread. (*A* and *C*) U2OS.TetR.HDAC4-FLAG cells were seeded at 1 × 10^6^ cells per well in six-well plates. After 24 h, cells were induced with doxycycline (100 ng/mL) for 24 h or left uninduced. Cells were then infected with VACV (*A*) or HSV-1 (*C*) at 0.0005 pfu per cell for 2 h, the inoculum was removed, and the infected cells were maintained in 1.5% carboxymethyl cellulose (CMC) in DMEM [+doxycycline (dox) where indicated]. Cells were fixed and stained with crystal violet after 3 (VACV) or 5 (HSV-1) d. Representative plaques are shown on the *Left*. Plaque size was quantified from 25 plaques in each condition. (*B* and *D*) U2OS.TetR.mcs (EV) and U2OS.TetR.HDAC4-FLAG cells were treated with dox (+) or mock treated (−) for 24 h and then infected with VACV or HSV-1 at 0.001 pfu per cell for 2 (VACV) or 3 (HSV-1) d. Supernatant and infected cells were collected and the infectious virus titer was determined by plaque assay on BSC-1 cells (VACV) and U2OS cells (HSV-1). Data shown are representative of three independent experiments. ns = not significant, *P < 0.05, ****P < 0.0001.

Given the role of HDAC4 in type I IFN signaling and its coprecipitation with STAT2, the effect of HDAC4 expression on the replication and spread of VACV and HSV-1 was investigated by gain-of-function and loss-of-function experiments. A U2OS cell line expressing an inducible FLAG-tagged HDAC4 was produced using a lentiviral vector (*Materials and Methods* and *SI Appendix*, Fig. S6). HDAC4 expression was then induced (+dox) or mock induced (−dox) and cells were infected with either VACV (Fig. 6 *A* and *B*) or HSV-1 (Fig. 6 *C* and *D*). Both the plaque size (Fig. 6 *A* and *C*) and virus titer (Fig. 6 *B* and *D*) of each virus were reduced by HDAC4 overexpression. In contrast, a control cell line transduced with the empty vector showed no difference in virus titer.

**Fig. 7.**
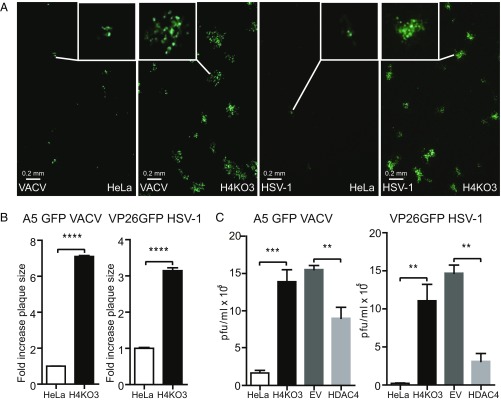
VACV and HSV-1 infection on HDAC4^−/−^ and HeLa cells. (*A*) HDAC4^−/−^ (H4KO3) and parental HeLa cells were seeded at 2 × 10^6^ cells per well in six-well plates. The number of cells for each cell line was measured before virus infection. Cells were infected with VACV A5GFP or HSV-1 VP26GFP infection at 0.001 pfu per cell for 3 h, washed twice with fresh medium, and maintained in DMEM with 2% FBS. After 2 (VACV) or 3 (HSV-1) d, images of infected cells were recorded with GFP excitation and emission spectra at 50-fold magnification. (*B*) Plaque size analysis. The sizes of plaques formed in *A* were quantified by AxioVision software (*n* = 20 per condition). (*C*) Virus replication. HeLa, H4KO3, H4KO3.mcs (EV), and H4KO3.HDAC4-FLAG (HDAC4) cells were infected with VACV or HSV-1 as in *A*. Infected cells and supernatants were collected 2 (VACV) or 3 (HSV-1) d after infection and the infectious virus titers were determined by plaque assay on BSC-1 cells (VACV) or U2OS cells (HSV-1). The cell monolayers were stained with crystal violet either 3 (VACV) or 5 (HSV-1) d after infection and plaque numbers were counted. Data shown are representative of three independent experiments. **P ≤ 0.01, ***P ≤ 0.001, ****P ≤ 0.0001.

The consequence of loss of HDAC4 was investigated next. Strains of VACV and HSV-1 that express GFP fused to virion proteins (A5GFP VACV and VP26GFP HSV-1) (45, 46) were used to infect HDAC4^−/−^ cells and the plaque sizes and virus titers were determined. The plaque size of both viruses increased substantially in HDAC4^−/−^ cells compared with HDAC4^+/+^ cells (Fig. 7 *A* and *B*). Similarly, yields of VACV and HSV-1 increased 9- or 400-fold, respectively, in HDAC4^−/−^ cells (Fig. 7*C*). Furthermore, transduction of HDAC4^−/−^ cells with FLAG-HDAC4–expressing lentivirus (47) reduced VACV and HSV-1 replication substantially compared with control cells transduced with empty vector (Fig. 7*C*). In summary, overexpression of HDAC4 reduced virus replication and loss of HDAC4 promoted virus replication, consistent with a role of HDAC4 as a viral restriction factor.

### HDAC4 Is Degraded During Vaccinia Virus Infection.

**Fig. 8.**
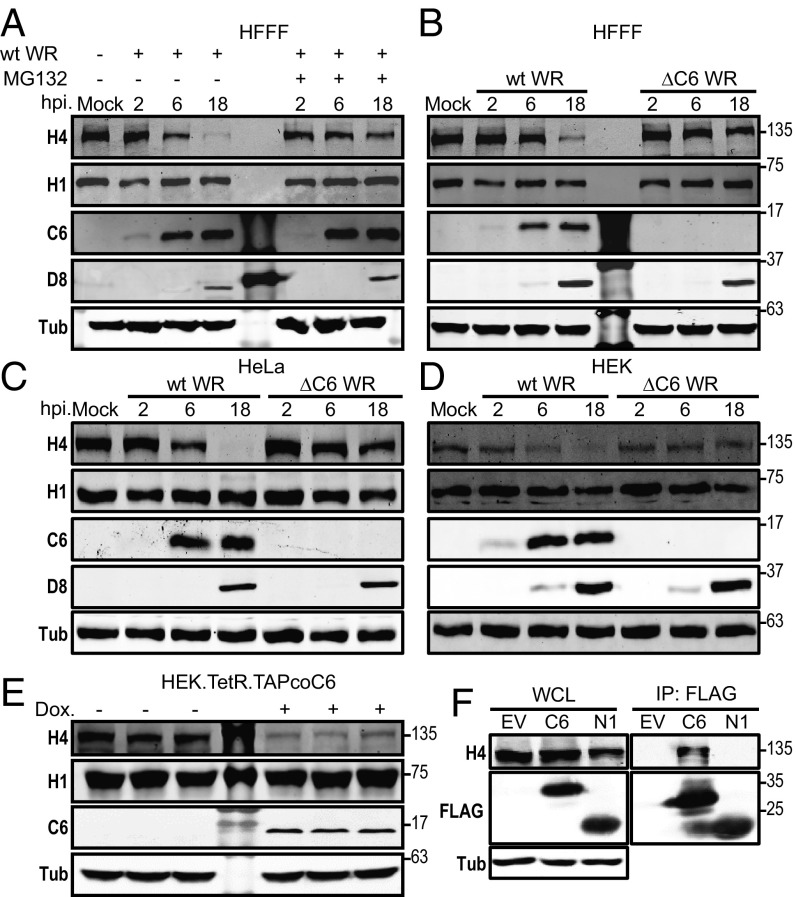
VACV induces proteasomal degradation of HDAC4 via protein C6. (*A*) HFFF-TERTs cells were infected with wild-type (wt) VACV strain WR, or mock infected. In parallel, infected cells were treated with 20 μM MG132 at 2 h p.i. At the indicated times, p.i. cell lysates were prepared and analyzed by immunoblotting with the indicated antibodies. (*B*) HFFF-TERTs cells were infected as in *A* with either wt VACV WR or a derivative mutant virus lacking the *C6L* gene (Δ6R). Lysates were prepared and analyzed by immunoblotting as in *A*. (*C* and *D*) VACV infection on HeLa and HEK-293T cells as in *B*. (*E*) Triplicate samples collected from HEK.TetR.TAPcoC6 cells either induced with 100 ng/mL doxycycline for 24 h or mock treated were analyzed by immunoblotting for HDAC4, HDAC1, TAPcoC6, and α-tubulin. (*F*) HEK-293T cells were transfected with empty vector (EV), TAP-tagged C6, or N1. After 24 h, cell lysates were prepared and precipitated via the strep-epitope. Inputs and immunoprecipitates were analyzed by immunoblotting for HDAC4, FLAG, and α-tubulin. The positions of molecular mass markers are shown in kilodaltons on the *Right*. Experiments in *A* and *B* were conducted four times and in *C*–*F* three times; representative images are shown. wt = wild type.

Viruses often evolve proteins to target host factors that restrict virus replication, either by neutralizing their biological activity or by inducing their degradation. To address if HDAC4 was stable during VACV infection, lysates from HFFF cells at different times p.i. were analyzed by immunoblotting (Fig. 8*A*). This showed that HDAC4 was down-regulated during infection, while HDAC1 remained stable. Further, addition of the proteasome inhibitor MG132 shortly after infection stabilized HDAC4 levels, demonstrating HDAC4 is targeted for proteasomal degradation. Given that HDAC4 is required for type I IFN signaling and coprecipitates with STAT2 via its TAD, and VACV protein C6 also has both these functions (36), we investigated if C6 was required for the degradation of HDAC4. Unlike WT VACV, infection with a VACV lacking the *C6L* gene (37) was unable to induce degradation of HDAC4 (Fig. 8*B*). To investigate whether the degradation of HDAC4 by C6 is cell line specific, the experiment was repeated in HeLa and HEK-293T cells, and this showed that HDAC4 was degraded and rescued by deletion of *C6L* gene (Fig. 8 *C* and *D*). Therefore, C6 induces degradation of HDAC4 in a proteasome-dependent manner. Finally, to determine if protein C6 was sufficient to induce degradation of HDAC4, a HEK-293T cell line expressing an inducible, codon-optimized, TAP-tagged C6 was constructed. Analysis of the levels of HDAC4 in these cells showed that induction of C6 expression alone caused degradation of HDAC4 (Fig. 8*E*). Therefore, C6 is necessary and sufficient for triggering the proteasomal degradation of HDAC4.

To start to understand how C6 might cause degradation of HDAC4, a possible interaction of C6 with HDAC4 was investigated by immunoprecipitation. TAP-tagged C6 and TAP-tagged VACV protein N1 were expressed in HEK-293T cells by transfection. C6, but not N1, coimmunoprecipitated with endogenous HDAC4. How this interaction between C6 and HDAC4 leads to the proteasomal degradation of HDAC4 remains to be determined, but a hypothesis to be examined in future is that C6 recruit components of the ubiquitin ligase system to induce ubiquitylation and consequential degradation of HDAC4.

## Discussion

This study reports that HDAC4 is required for type I IFN signaling, restricts the replication and spread of two large DNA viruses, and during VACV infection is targeted for proteasomal degradation via interaction with VACV protein C6.

Previous findings demonstrated roles for HDACs in either the production of type I IFN or the response to type I IFN (introduction). It was shown that pharmacological inhibition of HDACs by TSA reduced the response to type I IFN (16) and that HDAC1, a class I HDAC, was required for the IFN response (18). Given that TSA is a broad spectrum HDAC inhibitor (48), the possibility that other HDACs were required was investigated. The HDAC inhibitor LMK235, which preferentially inhibits HDAC5, HDAC4, and HDAC6 with IC_50_ values at 4.22, 11.9, and 55.7 nM, respectively (49), inhibited type I IFN signaling, suggesting a role of class II HDACs in type I IFN signaling. Knockout of HDAC5 caused lower STAT3 phosphorylation and transcriptional activity in leptin signaling (50). HDAC6 interacts with RIG-I during RNA virus infection and regulates the deacetylation of RIG-I and thereby promotes RIG-I sensing of viral RNAs leading to IFN-β expression (51). But neither HDAC5 nor HDAC6 have a known role in type I IFN signaling. In this study, the function of another class II HDAC, HDAC4, was investigated.

Pharmacological inhibition of HDAC activity by TSA, or of class II HDAC activity by LMK235, and knockout of HDAC4 in four independent cell lines, each caused a defective response to type I IFN using reporter gene assay and RT-qPCR of endogenous ISGs. This defect was rescued by reintroduction of HDAC4, but not HDAC5 or HDAC1. In addition, ChIP analysis of HDAC4 and STAT2 showed that HDAC4 is recruited to multiple ISG promoters following IFN-α stimulation, and HDAC4 is needed for the recruitment of STAT2 to ISG promoters. Given that HDACs are needed for IRF9-mediated recruitment of RNA polymerase II to ISG promoters (52), the ChIP analysis suggests that HDAC4 might regulate the assembly of the ISGF3 complex or the subsequent recruitment of RNA polymerase II. The HDAC-mediated alterations in nucleosome structure that correlate with H2A.Z occupancy following IFN-α stimulation (53) seem unlikely to be regulated by HDAC4 because HDAC4 enzymatic activity is not needed for type I IFN signaling.

Consistent with the possible regulation of ISGF3 assembly, HDAC4 coprecipitates with STAT2 via its transactivation domain, a property shared with VACV protein C6. Mutational analysis of HDAC4 showed that enzymatic activity of HDAC4 and its interaction with protein 14-3-3 were nonessential for type I IFN signaling and for interaction with STAT2. Given that the mutant HDAC4 3SA is located in the nucleus, HDAC4 has a nuclear function in this pathway. The inhibition of type I IFN signaling by LMK235, coupled with the observation that catalytically inactive HDAC4 functioned normally in type I IFN signaling, suggest another HDAC inhibited by LMK235 may be required. Consistent with this proposal, LMK235 inhibited type I IFN signaling in two HDAC4^−/−^ cell lines (*SI Appendix*, Fig. S5). LMK235 is most potent against HDAC5, but it inhibits several other class II HDACs. HDAC6 up-regulates IFN-β expression after stimulation with dsRNA (8) but its effect on type I IFN signaling is unknown. Therefore, it is possible that HDAC5, HDAC6, HDAC7, HDAC9, or HDAC10 might be also involved.

The biological relevance of HDAC4 in type I IFN signaling was demonstrated by analyzing the replication and spread of two large DNA viruses, VACV and HSV-1, in cells overexpressing HDAC4 and in HDAC4^−/−^ cells. Overexpression of HDAC4 in U2OS cells restricted the replication and spread of VACV and HSV-1 (Fig. 6). Conversely, in HDAC4^−/−^ cells, the replication and spread of both viruses were enhanced and this enhancement was restricted by the reintroduction of HDAC4 into these HDAC4^−/−^ cell lines (Fig. 7).

Recently, two other studies have reported the effect of HDAC4 knockdown or knockout. One study reported that following siRNA-induced reduction in HDAC4 there was enhanced phosphorylation of IRF3, leading to increased IFN-β expression (14). In agreement with this, using a reporter gene assay, we also found that overexpression of HDAC4 suppressed RIG-I– or TBK-1–induced activation of IRF3-dependent gene expression (*SI Appendix*, Fig. S7 *A* and *B*). The second study knocked out HDAC4 from Hep-2 cells and reported that this caused reduced HSV-1 replication (33). This contrasts with data presented here. However, the study used a different cell type to the several used here, and previously knockdown of STING from Hep-2 cells also caused a decrease in HSV-1 replication, whereas in two other cell types, STING knockdown enhanced virus replication (54). Also in Hep-2 cells, the authors did not complement the loss of HDAC4 activity, nor study the consequence of overexpression of HDAC4. Although HDAC4 seems to both inhibit IRF3 activation and activate type I IFN signaling, loss of HDAC4 enhanced HSV-1 and VACV replication and spread, whereas overexpression restricted these viruses, and so the dominant effect of HDAC4 activity is antiviral at least in HEK-293T, HeLa, and U2OS cells.

VACV encodes many antagonists of IFN production, signaling or ISG activity, reviewed in refs. 34 and 35. Here we show that VACV also induces the proteasomal degradation of HDAC4 during infection of several cell types (Fig. 8) and this requires protein C6, a virulence factor and multifunctional IFN antagonist (36, 37). C6 restricts the production of IFN-β by blocking activation of IRF-3 (37) and blocks type I IFN-induced JAK-STAT signaling (36). Like HDAC4, C6 coprecipitates with STAT2 via the C-terminal TAD (36). Herpesviruses also express proteins that antagonize IFN. For instance, HSV-1 proteins ICP27 and ICP0 inhibit type I IFN production or signaling (55–59), and ICP0 coprecipitates with HDAC4 (30). Similarly, EBNA-LP coprecipitates with HDAC4 (31). HDAC4 colocalizes with ND10, which is important in restricting HSV-1 infection (30, 60), and with ND10 members ATRX and SUMO (61, 62), consistent with a role for HDAC4 as a viral inhibitor in intrinsic immunity.

In summary, this study reports that HDAC4 is required for a normal response to type I IFN and that HDAC4^−/−^ cells are much more sensitive to VACV and HSV-1 infection, whereas the reintroduction of HDAC4 to HDAC4^−/−^ cells restricted viral replication. Thus, HDAC4 is a restriction factor for large DNA viruses. Consistent with this, HSV-1 and EBV encode proteins that coprecipitate with HDAC4 and may modify its function and, as we show here, HDAC4 is degraded during VACV infection by protein C6 and the proteasome. The targeting of this restriction factor by viruses emphasizes its biological importance against viruses.

## Materials and Methods

### Cell Lines.

Immortalized primary human fetal foreskin fibroblasts (HFFF-TERTs) (63), human HEK-293T, HeLa, U2OS (human osteosarcoma cell line), and BSC-1 (African green monkey cell line) cells were maintained in DMEM (Invitrogen) supplemented with 10% FBS (Pan Biotech) and penicillin/streptomycin (P/S, 50 μg/mL; Gibco). RK_13_ cells (rabbit kidney cell line) were maintained in MEM (Gibco) supplemented with 10% FBS and 50 μg/mL P/S.

### Plasmids and Viruses.

Plasmids used in this study were from the following sources: pcDNA3.HDAC4-FLAG (Addgene, 13821), pcDNA3.HDAC4 3SA-FLAG (Addgene, 30486), pcDNA3.HDAC5-FLAG (Addgene, 13822), and pcDNA3.HDAC1-FLAG (Addgene, 13820). pcDNA3.HA-STAT2, pcDNA3.HA-STAT1, pcDNA3.IRF9-TAP, pcDNA3.IRF9-S2C, pcDNA3.TAP-C6, and pcDNA3.N1-TAP were described (36). The reporter plasmids ISRE-luc, NF-κB–luc, or GAS-luc containing either ISRE, GAS, or NF-κB responsive promoters driving expression of firefly luciferase, and a plasmid with the thymidine kinase promoter driving expression of Renilla luciferase were kindly provided by Andrew Bowie, Trinity College, Dublin. ISG56.1-luc plasmid was a gift from Ganes Sen, Cleveland Clinic, Cleveland, OH. Lentivirus vector plasmids pLKOneo.EGFPnlsTetR and pLKO.DCMV.TetO.mcs (64) were gifts from Roger Everett, MRC, Centre for Virus Research, University of Glasgow, Glasgow, UK. pLKO.DCMV.TetO.HDAC4-FLAG and pLKO.DCMV.TetO.TAPcoC6 plasmids were constructed in the G.L.S. laboratory. Plasmids pCMV.dR8.91 (expressing all necessary lentivirus helper functions) and pMD-G (expressing the vesicular stomatitis virus envelope protein G) were obtained from Heike Laman, Department of Pathology, University of Cambridge, Cambridge, UK.

WT VACV strain Western Reserve (WR) and derivative strains expressing GFP fused to the capsid protein A5 (A5GFP VACV) (45), or lacking gene *C6L* were described (37). HSV-1 strain s17 expressing GFP fused to virus protein 26 (VP26GFP) was provided by Prashant Desai, Sidney Kummel Comprehensive Cancer Center, Johns Hopkins University, Baltimore, MD (46).

### Antibodies and Reagents.

The following antibodies were used: Rabbit (Rb) anti-HDAC4 (Cell Signaling, 2072), Rb anti-FLAG (Sigma-Aldrich, F7425), Rb anti-STAT2 (Santa Cruz, sc-476), Rb anti-STAT2 (Cell Signaling, 72604), Rb anti-STAT1 (Cell Signaling, 14994), Rb anti-C6 (described in ref. 37), mouse (Ms) anti–α-tubulin (Millipore, 05-829), Ms anti-HDAC1 (Santa Cruz, sc-81598), Ms anti-D8 (described in ref. 65), and Ms IgG (Invitrogen, 10400C). Secondary antibodies used were IRDye 800 goat anti-rabbit IgG (LICOR), and IRDye 800 donkey anti-mouse IgG (LICOR). The following reagents were used in ChIP assays: Ms anti-HDAC4 (Santa Cruz, sc-46672), Rb anti-STAT2 (Cell Signaling, 72604), Rb anti-STAT2 (ACTIVE MOTIF, 61651), Ms IgG (Sigma-Aldrich, I5381), Rb IgG (Sigma-Aldrich, I5006), Dynabeads Protein A (Invitrogen, 10002D), and Dynabeads Protein G (Invitrogen, 10004D). Proteasome inhibitor MG132 (Sigma-Aldrich, SML1135), HDAC inhibitor TSA (Tocris, 1406), and LMK235 (Tocris, 4830) were diluted in DMSO (Sigma-Aldrich, D8418). Plasmids were transfected using TransIT-LT1 transfection reagent (Mirus, MIR 2306). IFN-α (300-02AA), IFN-γ (300-02), TNF-α (300-01A), and IL-1β (200-01B) were all from Peprotech. Immunoprecipitation was performed with ANTI-FLAG M2 Affinity Gel (Sigma-Aldrich, A2220), Pierce High Capacity Streptavidin Agarose (Thermo Fisher Scientific, 20357), or Protein G Sepharose (GE Healthcare, 17-0618-01).

### Lentivirus Transductions.

HEK-293T cells (3 × 10^6^) were seeded in a 10-cm dish on day 1. On day 2, the cells were transfected with 3 μg pLKOneo.EGFPnlsTetR together with 3 μg of each pMD-G and pCMV.dR8.91 plasmids. Three hours posttransfection, the cell culture medium was removed and replaced with DMEM with 30% FBS and 50 μg/mL P/S. On day 3, the cell culture supernatant was collected and replaced with 5 mL DMEM containing 30% FBS. The collected supernatant was passed through a 0.45-μm filter and 2 μg/mL Polybrene (Sigma-Aldrich, H9268) was added. This lentivirus stock was used to infect U2OS cells that were seeded on day 2. On day 4, the same lentivirus infection was repeated. Following the lentivirus infection, transduced cells were selected with 500 µg/mL neomycin (BioVision, 1557), and thereafter neomycin was maintained in the transduced cell culture medium. The same method was used to prepare HDAC4-FLAG expression lentivirus with plasmid pLKO.TetO.HDAC4-FLAG. Following the HDAC4-FLAG expression lentivirus infection, cells were selected with 1 µg/mL puromycin (InvivoGen, 58-58-2).

HDAC4^−/−^ cell lines derived from either HeLa or HEK-293T cells were selected as described in the legend to *SI Appendix*, Fig. S2. HDAC4^−/−^ HeLa clone H4KO3 cells stably expressing HDAC4-FLAG or transduced with empty vector cells were obtained by transduction with lentiviruses and selected with puromycin (1 µg/mL).

### Immunoblotting.

Cells were lysed in passive lysis buffer (Promega, E194A) and lysates were boiled at 98 °C for 5 min. The samples were cooled to room temperature and the insoluble debris was collected by centrifugation at (17,000 × *g*) for 1 min. Samples were then analyzed by SDS-polyacrylamide gel electrophoresis (PAGE) alongside protein molecular mass markers (Abcam, ab116028). Proteins separated by SDS/PAGE were transferred onto nitrocellulose membranes (GE Healthcare) by using Trans-Blot Turbo transfer system (Bio-Rad) in 25 mM Tris⋅HCl, 250 mM glycine, and 20% methanol. Membranes were blocked with 5% skimmed milk in PBST or 5% BSA in TBST at 4 °C overnight. Blocked membranes were then incubated with primary antibodies overnight at 4 °C with consistent agitation, washed three times in PBST or TBST, and then incubated with secondary antibodies for 2 h at room temperature. The membranes were then washed three times with PBST or TBST, dried at room temperature, and imaged using the LI-COR Odyssey imaging system.

### Reporter Gene Assay.

HEK-293T, HeLa, or derivative HDAC4^−/−^ cells were seeded into 96-well plates at 4 × 10^4^ cells per well and the next day transfected with 100 ng of either ISRE-luc or NF-κB–luc, together with 10 ng TK-Renilla luciferase. Complementation assays on HDAC4^−/−^ cells were carried out by transfection of ISRE-luc, TK-Renilla, and either HDAC4-FLAG, HDAC5-FLAG, HDAC1-FLAG, or HDAC4 3SA-FLAG expressing plasmids. Following overnight incubation, transfected cells were stimulated by 1,000 units/mL IFN-α or 10 ng/mL TNF-α for 8 h. The cell culture medium was removed after cytokine stimulation and the cells were lysed with 100 μL passive lysis buffer (Promega, E1910). The cell lysate was kept at −20 °C and the firefly and Renilla luciferase activity were measured within 2 wk. To measure the luciferase activity, 50 μL firefly luciferase reagent [20 mM tricine, 2.67 mM MgSO_4_⋅7H_2_O, 0.1 mM EDTA, 33.3 mM DTT, 530 μM ATP, 270 μM acetyl-CoA, 132 μg/mL luciferin (Prolume), 5 mM NaOH, 0.26 mM MgCO_3_Mg(OH)_2_⋅5H_2_O], or 50 μL Renilla luciferase reagent (2 μg/mL coelenterazine in PBS) (Nanolight Technology, 350-10) was added to 10 μL cell lysate. The luminescence value was measured with a microplate reader (BMG Labtech). Firefly luciferase values were normalized to the Renilla luciferase values and the fold inductions in each reporter gene assay were calculated relative to the unstimulated controls. Experiments were performed in triplicate and conducted at least three times.

### Chromatin Immunoprecipitation Assay.

HeLa and H4KO3 cells were seeded at 1 × 10^8^ cells for each condition (with/without IFN-α treatment). Cells were stimulated or mock treated with IFN-α (1,000 units/mL) for 3 h and processed as described with slight modification (66). Briefly, cells were fixed with 1% formaldehyde for 10 min and sonicated with a Bioruptor Pico sonication device (Diagenode, B01060010) (13 cycles of 30 s each at 22% of maximum amplitude). For each ChIP condition, 7 µg of antibodies against HDAC4, STAT2, or IgG isotype control were incubated with the sonicated chromatin overnight at 4 °C. The subsequent immune complexes were enriched by a mixture of Dynabeads Protein G and A (1:1) with a 3-h incubation at 4 °C. The samples were washed four times and decrosslinked at 68 °C overnight with constant agitation in ChIP elution buffer. Enriched DNA was purified with Qiagen PCR purification kit (QIAGEN, 28106). The ChIPs were validated at STAT2 target ISGs (*IFIT1, IFIT3*, and *ISG15*) by qPCR. Primer sequences used in the above qPCR were reported (53) previously.

### RT-qPCR.

Cells (2 × 10^6^) were seeded on six-well plates 1 d before stimulation with 1,000 units/mL IFN-α or 100 ng/mL interleukin (IL)-1β for 4 h. The cells were harvested and the total mRNA was extracted using the RNeasy Mini kit (Qiagen) according to the manufacturer’s instructions. Thereafter, 500 ng of RNA was used to synthesize cDNA using SuperScript III reverse transcriptase (Invitrogen).

RT-qPCR analysis of the level of mRNA for specific ISGs was performed on HDAC4^+/+^ and HDAC4^−/−^ HeLa cells. Each individual reaction was performed in duplicate, using SYBR Green Master Mix, following the manufacturer’s protocol (Thermo Fisher Scientific, 4309155). ViiA 7 real-time PCR system (Thermo Fisher Scientific) was used to determine the cycle threshold of each reaction and determine the fold induction of the investigated genes. Gene amplification was normalized to *GAPDH* amplification and the fold induction was determined relative to control cells that had not been stimulated with IFN-α.

### Virus Infection and Fluorescent Microscopy.

WT VACV strain WR and derivative strains expressing GFP fused to the capsid protein A5 (A5GFP VACV) (45) or lacking gene *C6L* were described (37). VACV strains were grown on RK_13_ cells and titrated by plaque assay on BSC-1 cells. HSV-1 strain s17 expressing GFP fused to virus protein 26 (VP26GFP) was provided by Prashant Desai (46). This virus was grown and titrated on U2OS cells. To measure virus plaque size, monolayers of HeLa cells or H4KO3 cells were infected with 20–50 pfu per well and after 3 d the size of virus plaques (*n* = 20) was measured at 50× magnification using AxioVision 4.8 software and a ZEISS Axio Vert.A1 fluorescent microscope.

To measure virus replication, monolayers of HeLa or H4KO3 were infected at 0.001 pfu per cell and the yield of infectious total virus present at 2 d (A5GFP VACV) or 3 d (VP26GFP HSV-1) postinfection was determined by plaque assay on BSC-1 cells or U2OS cells for VACV and HSV-1, respectively. Measurements were made from multiple independent experiments (*n* = 4).

### Statistical Analysis.

Unpaired Student’s *t* tests were performed using the statistics module from GraphPad PRISM 5.0. Welch’s correction was applied where variance was shown to be significant. Statistical significance is expressed as follows: not significant (NS), *P* > 0.05, *P < 0.05, **P < 0.01, ***P < 0.001, ****P < 0.0001.

## Supplementary Material

Supplementary File
