# Supplementary material for: Histone deacetylase 4 promotes type I interferon signaling, restricts DNA viruses, and is degraded via vaccinia virus protein C6
Source: Proc Natl Acad Sci U S A. 2019 May 24;116(24):11997–2006. doi: 10.1073/pnas.1816399116 (PMC6575207; doi:10.1073/pnas.1816399116)
Supplement: Supplementary File [file pnas.1816399116.sapp.pdf]

Supplementary Information for

Histone deacetylase 4 promotes type I interferon signaling, restricts DNA virus replication and is degraded via vaccinia virus protein C6

Yongxu Lu, Jennifer H. Stuart**, Callum Talbot-Cooper, Shuchi Agrawal-Singh†, Brian Huntly†, Andrei I. Smid, Joseph S. Snowden[†], Liane Dupon & Geoffrey L. Smith*

Department of Pathology, University of Cambridge, Tennis Court Road, Cambridge, CB2 1QP United Kingdom

*Communicating author: gls37@cam.ac.uk

† Cambridge Institute for Medical Research, University of Cambridge, Hills Road, Cambridge, CB2 0XY, UK

[†] Current address: Astbury Centre for Structural Molecular Biology, University of Leeds, Leeds, LS2 9JT, UK. **Current address: Global Health Security Programme, Department of Health, 39 Victoria Street, London, SW1H 0EU, UK.

This PDF file includes:

Figs. S1 to S7
Reference for SI reference citation

Supplementary Figures

www.pnas.org/cgi/doi/10.1073/pnas.1816399116

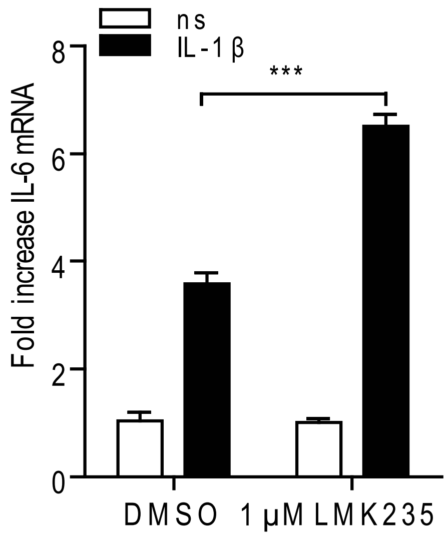

Fig. S1. The HDAC inhibitor LMK235 does not inhibit the response to IL-1β. HeLa cells were treated simultaneously with 1 μM LMK235 or DMSO and 100 ng/ml IL-1β for 6 h. mRNA was extracted from cells and used for RT-qPCR analysis. Data are presented as the fold induction of IL-6 mRNA expression relative to the unstimulated, DMSO-treated control and relative to GAPDH mRNA expression. Data shown are representative of three independent experiments. (***$p \leqslant 0.001$).

A

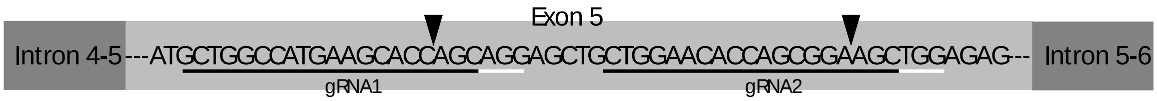

B

Primer	sequence
HDAC4 Exon5 Forward	GTGTCCACCTTCGAGGGAAC
HDAC4 Exon5 Reverse	TGACACACTGTCACACTCAC

C

Cell lines	Genomic DNA sequence of HDAC4 Exon5	Allele	No. of sequences
HEK293T	CAACAACAGGAGATGCTGGCCATGAAGCACCAGCAGGAGCTGCTGGAACACCAGCGGAAGCTGGAGAGG		
H4KO1	CAACAACAGGAGATGCTGGCCATGAAGCACCAGCAGGAGCTGCTGGAACACCAGCGG-------AGAGG	1	10
	CAACAACAGGAGATGCTGGCCATGAAGCACCAGCAGGAGCTGCTGGAACACCAGCGGA-GCTGGAGAGG	2	7
H4KO2	CAACAACAGGAGATGCTGGCCA-------------------------------GCGGAAGCTGGAGAGG	1	6
	CAACAACAGGAGATGCTGGCCATGAAGCAC-AGCAGGAGCTGCTGGAACACCAGCGGAAGCTGGAGAGG	2	11

Cell lines	Genomic DNA sequence of HDAC4 Exon5	Allele	No. of sequences
HeLa	CAACAACAGGAGATGCTGGCCATGAAGCACCAGCAGGAGCTGCTGGAACACCAGCGGAAGCTGGAGAGG		
H4KO3	CAACAACAGGAGATGCTGGCCATGAAGCAC-AGCAGGAGCTGCTGGAACACCAGCGGAAGCTGGAGAGG	1	5
	CAACAACAGGAGATGCTGGCCATGAA----CAGCAGGAGCTGCTGGAACGCCAGCGGAAGCTGGAGAGG	2	10
H4KO4	CAACAACAGGAGATGCTGGCCATGAAGCAC-AGCAGGAGCTGCTGGAACACCAGCGGAAGCTGGAGAGG	1	5
	CAACAACAGGAGATGCTGGCCATGAAGCACCCGAGCAGGAGCTGCTGGAACACCAGCGGAAGCTGGAGAGG	2	7

Fig. S2. CRISPR/Cas9-mediated genome editing of HDAC4 in HEK-293T and HeLa cells. Human HDAC4 has 7 splice forms, with the largest containing 27 exons and encoding a 1084 amino acid protein. (A) Two guide RNAs (gRNAs) were designed using online software (1) to target HDAC4 exon 5, which is shared between all HDAC4 splice forms (gRNA sequence underlined. PAM sequence underlined in white). px459 CRISPR/Cas9 plasmids with or without gRNA sequence were transfected into HEK-293T and HeLa cells. Puromycin-resistant cells were selected and individual clones were obtained after serial dilution. Several clones from each cell type were then amplified. (B, C) Genomic DNA from potential knockout clones was then sequenced at the gRNA target sites. For each cell line, a PCR-amplified DNA fragment containing exon 5 was purified, cloned into bacterial plasmids and from each bacterial transformation, multiple clones (*n* = 20-40) were sequenced. These all contained frameshift mutations and an absence of the wild type allele.

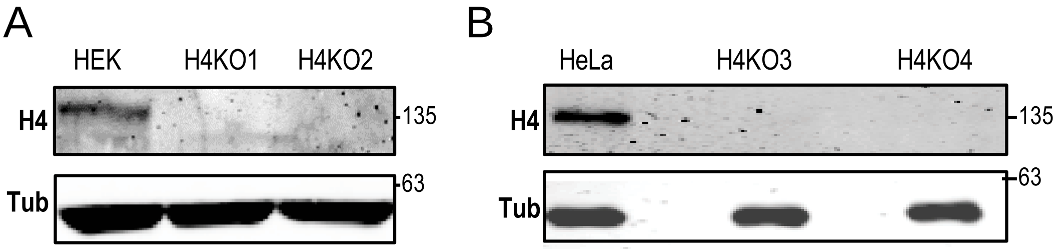

Fig. S3. Immunoblot analysis of HDAC4$^{-/-}$ clones derived from HEK-293T (H4KO1, H4KO2) and HeLa (H4KO3 and H4KO4). Cell lysates were collected from 1×10^7 cells of each cell line. Cells were incubated with 1 ml cell lysis buffer (PBS+1% NP40) with constant agitation at 4 °C. After 6 h incubation, the cell lysates were cleared by centrifugation and the supernatants were diluted with equal amounts of 2 × western blot loading buffer (125 mM Tris-Cl, pH 6.8, 4% (w/v) sodium dodecyl sulfate (SDS), 0.02% (w/v) bromophenol blue, 20% (v/v) glycerol and 200 mM dithiothreitol (DTT)) for immune blotting analysis. Rabbit anti-HDAC4 was used to measure HDAC4 expression and a mAb anti-α-tubulin was used to indicate equal cell populations were used in the assay. The images shown are representative of three independent experiments.

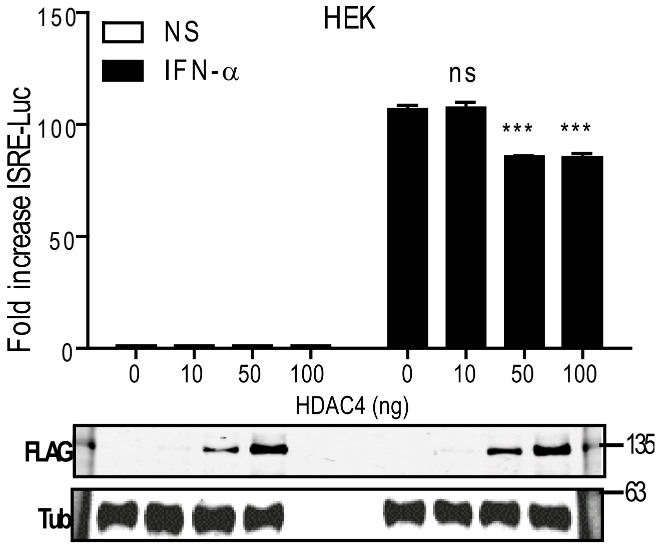

Fig. S4. ISRE-luciferase reporter gene assay in HEK-293T cells expressing FLAG-tagged HDAC4. ISRE-luciferase, renilla and HDAC4-FLAG expression plasmids were co-transfected into HEK-293T cells overnight. The HDAC4-FLAG expression plasmid was transfected at different doses as indicated. Cells were stimulated by 1000 unit/ml IFN-α for 6 h and cell lysates were collected and firefly and renilla luciferase expression was measured. Firefly luciferase was normalised to renilla and the fold induction relative to unstimulated controls is shown. The bottom panel shows an immunoblot for HDAC4-FLAG and α-tubulin (Tub) as loading control. Data shown are representative of three independent experiments. (***$p \leqslant 0.001$).

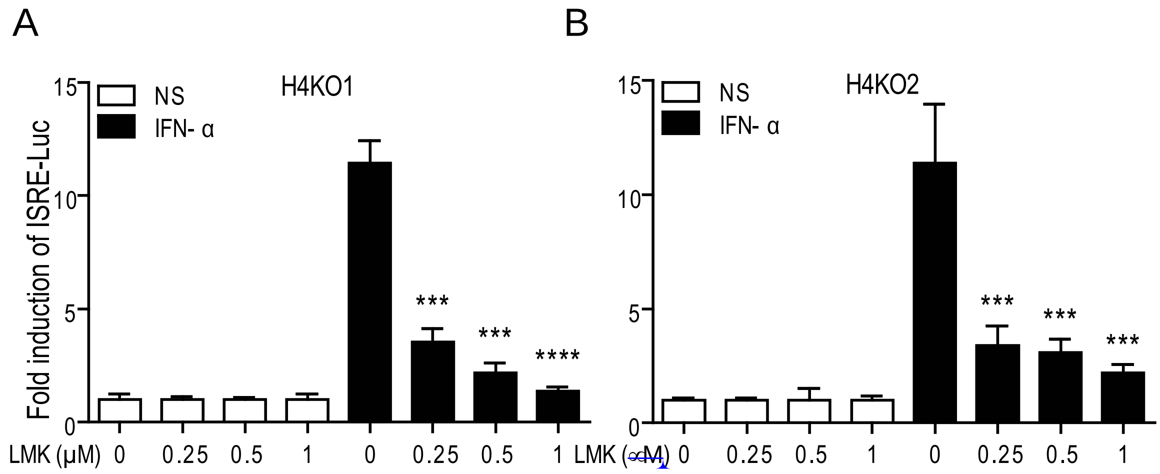

Fig. S5. The HDAC inhibitor LMK235 inhibits the IFN-α response in HDAC4 $^{-/-}$ cells. H4KO1 and H4KO2 cells were co-transfected with ISRE-luciferase and Renilla-luciferase plasmids as described in Fig. 2 (A). Transfected cells were treated simultaneously with different doses of LMK235 as indicated and 1000 unit/ml IFN-α for 6 h. The cell lysates were collected and analyzed as described in Fig. 2 (A). Data shown are representative of three independent experiments. (***p⩽0.001, ****p⩽0.0001).

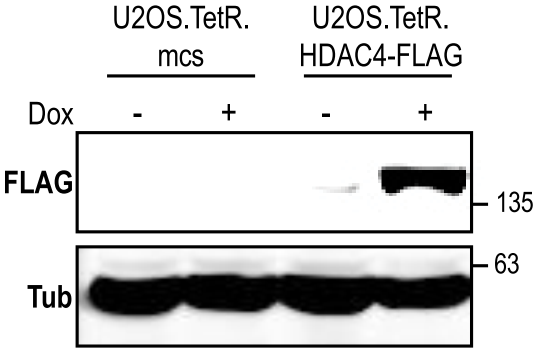

Fig. S6. U2OS cells expressing HDAC4-FLAG. Stocks of lentivirus vector expressing HDAC4-FLAG or the empty vector (mcs) were transduced into U2OS.TetR cells that express nlsTetR-EGFP fusion protein in nucleus. U2OS.TetR.mcs or U2OS.TetR.HDAC4-FLAG cells (2×10^6 cells) were seeded in 6-well plates. The following day cells were induced with 100 ng/ml doxycycline (+ dox) or left untreated (-) and 24 h later HDAC4-FLAG and α -tubulin expression were analyzed by immunoblot using monoclonal antibody (mAb) anti-FLAG and anti-α-tubulin. The positions of molecular mass markers are indicated in kDa. The image shown is representative of three independent experiments.

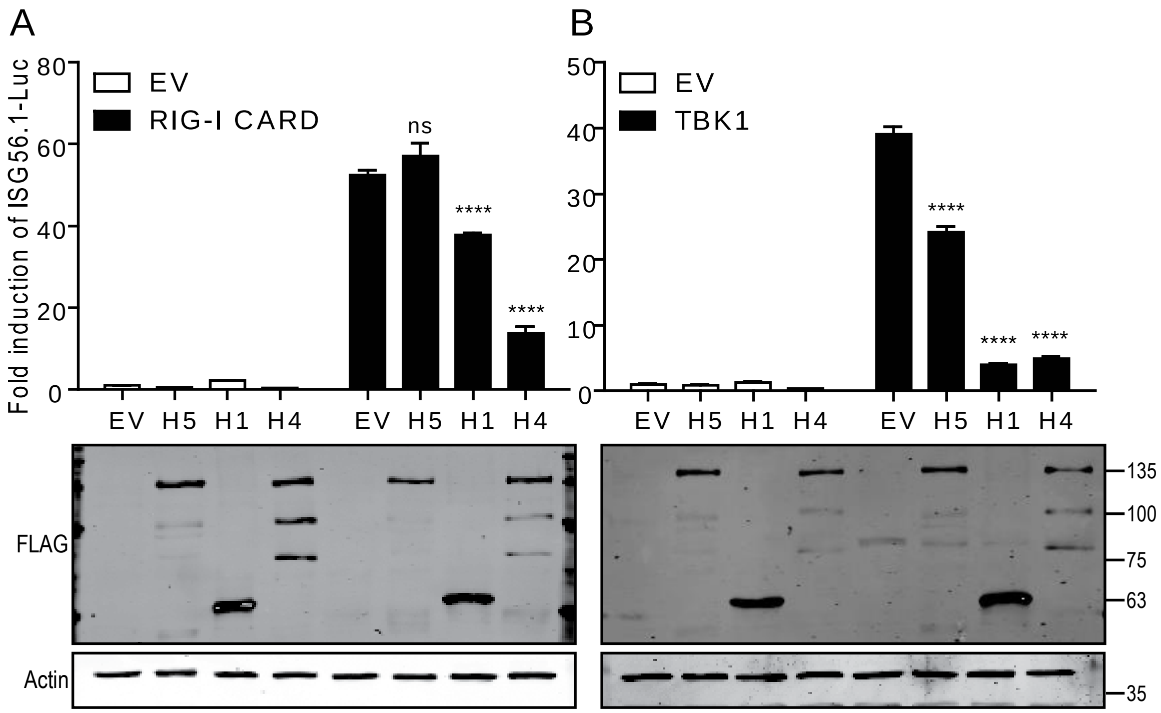

Fig. S7. HDAC4 downregulates RIG-I-CARD and TBK1-stimulated IRF3 activation. (A) HEK-293T cells in 96-well plates were transfected with 100 ng of ISG56.1-firefly luciferase reporter plasmid, 10 ng of renilla luciferase control plasmid, 50 ng of HDAC1 (H1), HDAC4 (H4) or HDAC5 (H5) expression plasmid or empty vector (EV) and either 5 ng of RIG-I- caspase recruitment domains (CARD) expression plasmid or EV per well. After 24 h, the cells were harvested in passive lysis buffer and the firefly luciferase activity was measured and normalised to the renilla luciferase activity. Bottom panel: Immunoblot showing the expression of HDAC1-FLAG, HDAC4-FLAG, HDAC5-FLAG and actin as loading control. (B) As in (A) except that 20 ng/well TBK-1 expression plasmid was used to activate the IRF3 pathway. Data shown are representative of three experiments, each performed with six replicates per experimental condition. (****$p \leqslant 0.01$.).

References

1. Ran FA, *et al.* (2013) Genome engineering using the CRISPR-Cas9 system. *Nat Protoc* 8(11):2281-2308.
